# The sequence of sequencers: The history of sequencing DNA

**DOI:** 10.1016/j.ygeno.2015.11.003

**Published:** 2016-01

**Authors:** James M. Heather, Benjamin Chain

**Affiliations:** Division of Infection & Immunity, UCL Cruciform Building, Gower Street, London, United Kingdom

**Keywords:** DNA, RNA, Sequencing, Sequencer, History

## Abstract

Determining the order of nucleic acid residues in biological samples is an integral component of a wide variety of research applications. Over the last fifty years large numbers of researchers have applied themselves to the production of techniques and technologies to facilitate this feat, sequencing DNA and RNA molecules. This time-scale has witnessed tremendous changes, moving from sequencing short oligonucleotides to millions of bases, from struggling towards the deduction of the coding sequence of a single gene to rapid and widely available whole genome sequencing. This article traverses those years, iterating through the different generations of sequencing technology, highlighting some of the key discoveries, researchers, and sequences along the way.

## Highlights

• We review the drastic changes to DNA sequencing technology over the last 50 years.
• First-generation methods enabled sequencing of clonal DNA populations.
• The second-generation massively increased throughput by parallelizing many reactions.
• Third-generation methods allow direct sequencing of single DNA molecules.

## 1 Introduction

“*...* [*A*] *knowledge of sequences could contribute much to our understanding of living matter.*”

Frederick Sanger [1]

The order of nucleic acids in polynucleotide chains ultimately contains the information for the hereditary and biochemical properties of terrestrial life. Therefore the ability to measure or infer such sequences is imperative to biological research. This review deals with how researchers throughout the years have addressed the problem of how to sequence DNA, and the characteristics that define each generation of methodologies for doing so.

## 2 First-generation DNA sequencing

Watson and Crick famously solved the three-dimensional structure of DNA in 1953, working from crystallographic data produced by Rosalind Franklin and Maurice Wilkins [2], [3], which contributed to a conceptual framework for both DNA replication and encoding proteins in nucleic acids. However, the ability to ‘read’ or sequence DNA did not follow for some time. Strategies developed to infer the sequence of protein chains did not seem to readily apply to nucleic acid investigations: DNA molecules were much longer and made of fewer units that were more similar to one another, making it harder to distinguish between them [4]. New tactics needed to be developed.

Initial efforts focused on sequencing the most readily available populations of relatively pure RNA species, such as microbial ribosomal or transfer RNA, or the genomes of single-stranded RNA bacteriophages. Not only could these be readily bulk-produced in culture, but they are also not complicated by a complementary strand, and are often considerably shorter than eukaryotic DNA molecules. Furthermore, RNase enzymes able to cut RNA chains at specific sites were already known and available. Despite these advantages, progress remained slow, as the techniques available to researchers – borrowed from analytical chemistry – were only able to measure nucleotide composition, and not order [5]. However, by combining these techniques with selective ribonuclease treatments to produce fully and partially degraded RNA fragments [6] (and incorporating the observation that RNA contained a different nucleotide base [7]), in 1965 Robert Holley and colleagues were able to produce the first whole nucleic acid sequence, that of alanine tRNA from *Saccharomyces cerevisiae* [8]. In parallel, Fred Sanger and colleagues developed a related technique based on the detection of radiolabelled partial-digestion fragments after two-dimensional fractionation [9], which allowed researchers to steadily add to the growing pool of ribosomal and transfer RNA sequences [10], [11], [12], [13], [14]. It was also by using this 2-D fractionation method that Walter Fiers' laboratory was able to produce the first complete protein-coding gene sequence in 1972, that of the coat protein of bacteriophage MS2 [15], followed four years later by its complete genome [16].

It was around this time that various researchers began to adapt their methods in order to sequence DNA, aided by the recent purification of bacteriophages with DNA genomes, providing an ideal source for testing new protocols. Making use of the observation that Enterobacteria phage *λ* possessed 5′ overhanging ‘cohesive’ ends, Ray Wu and Dale Kaiser used DNA polymerase to fill the ends in with radioactive nucleotides, supplying each nucleotide one at a time and measuring incorporation to deduce sequence [17], [18]. It was not long before this principle was generalized through the use of specific oligonucleotides to prime the DNA polymerase. Incorporation of radioactive nucleotides could then be used to infer the order of nucleotides anywhere, not just at the end termini of bacteriophage genomes [19], [20], [21]. However the actual determination of bases was still restricted to short stretches of DNA, and still typically involved a considerable amount of analytical chemistry and fractionation procedures.

**Fig. 1:**
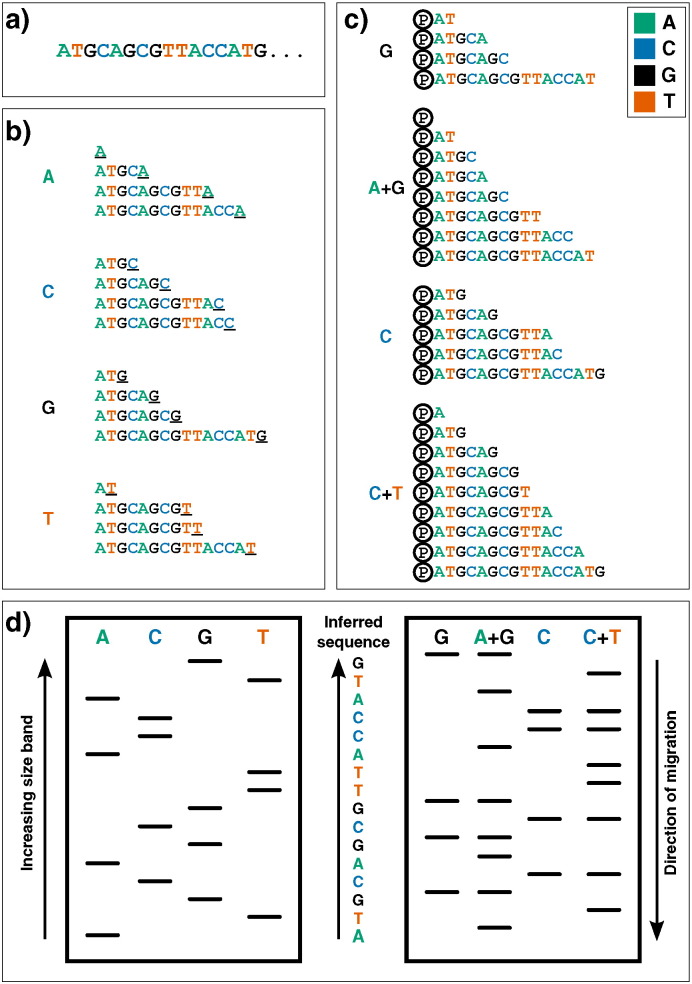
First-generation DNA sequencing technologies. Example DNA to be sequenced **(**a) is illustrated undergoing either Sanger (b) or Maxam–Gilbert (c) sequencing. (b): Sanger's ‘chain-termination’ sequencing. Radio- or fluorescently-labelled ddNTP nucleotides of a given type - which once incorporated, prevent further extension - are included in DNA polymerisation reactions at low concentrations (primed off a 5′ sequence, not shown). Therefore in each of the four reactions, sequence fragments are generated with 3′ truncations as a ddNTP is randomly incorporated at a particular instance of that base (underlined 3′ terminal characters). (c): Maxam and Gilbert's ‘chemical sequencing’ method. DNA must first be labelled, typically by inclusion of radioactive P ^32^ in its 5′ phosphate moiety (shown here by Ⓟ). Different chemical treatments are then used to selectively remove the base from a small proportion of DNA sites. Hydrazine removes bases from pyrimidines (cytosine and thymine), while hydrazine in the presence of high salt concentrations can only remove those from cytosine. Acid can then be used to remove the bases from purines (adenine and guanine), with dimethyl sulfate being used to attack guanines (although adenine will also be affected to a much lesser extent). Piperidine is then used to cleave the phophodiester backbone at the abasic site, yielding fragments of variable length. (d): Fragments generated from either methodology can then be visualized via electrophoresis on a high-resolution polyacrylamide gel: sequences are then inferred by reading ‘up’ the gel, as the shorter DNA fragments migrate fastest. In Sanger sequencing (left) the sequence is inferred by finding the lane in which the band is present for a given site, as the 3′ terminating labelled ddNTP corresponds to the base at that position. Maxam–Gilbert sequencing requires a small additional logical step: Ts and As can be directly inferred from a band in the pyrimidine or purine lanes respectively, while G and C are indicated by the presence of dual bands in the G and A + G lanes, or C and C + T lanes respectively.

The next practical change to make a large impact was the replacement of 2-D fractionation (which often consisted of both electrophoresis and chromatography) with a single separation by polynucleotide length via electrophoresis through polyacrylamide gels, which provided much greater resolving power. This technique was used in two influential yet complex protocols from the mid-1970s: Alan Coulson and Sanger's ‘plus and minus’ system in 1975 and Allan Maxam and Walter Gilbert's chemical cleavage technique [22], [23]. The plus and minus technique used DNA polymerase to synthesize from a primer, incorporating radiolabelled nucleotides, before performing two second polymerisation reactions: a ‘plus’ reaction, in which only a single type of nucleotide is present, thus all extensions will end with that base, and a ‘minus’ reaction, in which three are used, which produces sequences up to the position before the next missing nucleotide. By running the products on a polyacrylamide gel and comparing between the eight lanes, one is able to infer the position of nucleotides at each position in the covered sequence (except for those which lie within a homopolymer, i.e. a run of the same nucleotide). It was using this technique that Sanger and colleagues sequenced the first DNA genome, that of bacteriophage *ϕ*X174 (or ‘PhiX’, which enjoys a position in many sequencing labs today as a positive control genome) [24]. While still using polyacrylamide gels to resolve DNA fragments, the Maxam and Gilbert technique differed significantly in its approach. Instead of relying on DNA polymerase to generate fragments, radiolabelled DNA is treated with chemicals which break the chain at specific bases; after running on a polyacrylamide gel the length of cleaved fragments (and thus position of specific nucleotides) can be determined and therefore sequence inferred (see Fig. 1, right). This was the first technique to be widely adopted, and thus might be considered the real birth of ‘first-generation’ DNA sequencing.

However the major breakthrough that forever altered the progress of DNA sequencing technology came in 1977, with the development of Sanger's ‘chain-termination’ or dideoxy technique [25]. The chain-termination technique makes use of chemical analogues of the deoxyribonucleotides (dNTPs) that are the monomers of DNA strands. Dideoxynucleotides (ddNTPs) lack the 3′ hydroxyl group that is required for extension of DNA chains, and therefore cannot form a bond with the 5′ phosphate of the next dNTP [26]. Mixing radiolabelled ddNTPs into a DNA extension reaction at a fraction of the concentration of standard dNTPs results in DNA strands of each possible length being produced, as the dideoxy nucleotides get randomly incorporated as the strand extends, halting further progression. By performing four parallel reactions containing each individual ddNTP base and running the results on four lanes of a polyacrylamide gel, one is able to use autoradiography to infer what the nucleotide sequence in the original template was, as there will a radioactive band in the corresponding lane at that position of the gel (see Fig. 1, left). While working on the same principle as other techniques (that of producing all possible incremental length sequences and labelling the ultimate nucleotide), the accuracy, robustness and ease of use led to the dideoxy chain-termination method – or simply, Sanger sequencing – to become the most common technology used to sequence DNA for years to come.

A number of improvements were made to Sanger sequencing in the following years, which primarily involved the replacement of phospho- or tritrium-radiolabelling with fluorometric based detection (allowing the reaction to occur in one vessel instead of four) and improved detection through capillary based electrophoresis. Both of these improvements contributed to the development of increasingly automated DNA sequencing machines [27], [28], [29], [30], [31], [32], [33], and subsequently the first crop of commercial DNA sequencing machines [34] which were used to sequence the genomes of increasingly complex species.

These first-generation DNA sequencing machines produce reads slightly less than one kilobase (kb) in length: in order to analyse longer fragments researchers made use of techniques such as ‘shotgun sequencing’ where overlapping DNA fragments were cloned and sequenced separately, and then assembled into one long contiguous sequence (or ‘contig’) in silico [35], [36]. The development of techniques such as polymerase chain reaction (PCR) [37], [38] and recombinant DNA technologies [39], [40] further aided the genomics revolution by providing means of generating the high concentrations of pure DNA species required for sequencing. Improvements in sequencing also occurred by less direct routes. For instance, the Klenow fragment DNA polymerase – a fragment of the *Escherichia coli* DNA polymerase that lacks 5′ to 3′ exonuclease activity, produced through protease digestion of the native enzyme [41] – had originally been used for sequencing due to its ability to incorporate ddNTPs efficiently. However, more sequenced genomes and tools for genetic manipulation provided the resources to find polymerases that were better at accommodating the additional chemical moeities of the increasingly modified dNTPs used for sequencing [42]. Eventually, newer dideoxy sequencers – such as the ABI PRISM range developed from Leroy Hood's research, produced by Applied Biosystems [43], which allowed simultaneous sequencing of hundreds of samples [44] – came to be used in the Human Genome Project, helping to produce the first draft of that mammoth undertaking years ahead of schedule [45], [46].

## 3 Second-generation DNA sequencing

Concurrent with the development of large-scale dideoxy sequencing efforts, another technique appeared that set the stage for the first wave in the next generation of DNA sequencers. This method markedly differed from existing methods in that it did not infer nucleotide identity through using radio- or fluorescently-labelled dNTPs or oligonucleotides before visualising with electrophoresis. Instead researchers utilized a recently discovered luminescent method for measuring pyrophosphate synthesis: this consisted of a two-enzyme process in which ATP sulfurylase is used to convert pyrophosphate into ATP, which is then used as the substrate for luciferase, thus producing light proportional to the amount of pyrophosphate [47]. This approach was used to infer sequence by measuring pyrophosphate production as each nucleotide is washed through the system in turn over the template DNA affixed to a solid phase [48]. Note that despite the differences, both Sanger's dideoxy and this pyrosequencing method are ‘sequence-by-synthesis’ (SBS) techniques, as they both require the direct action of DNA polymerase to produce the observable output (in contrast to the Maxam–Gilbert technique). This pyrosequencing technique, pioneered by Pål Nyrén and colleagues, possessed a number of features that were considered beneficial: it could be performed using natural nucleotides (instead of the heavily-modified dNTPs used in the chain-termination protocols), and observed in real time (instead of requiring lengthy electrophoreses) [49], [50], [51]. Later improvements included attaching the DNA to paramagnetic beads, and enzymatically degrading unincorporated dNTPs to remove the need for lengthy washing steps. The major difficulty posed by this technique is finding out how many of the same nucleotide there are in a row at a given position: the intensity of light released corresponds to the length of the homopolymer, but noise produced a non-linear readout above four or five identical nucleotides [51]. Pyrosequencing was later licensed to 454 Life Sciences, a biotechnology company founded by Jonathan Rothburg, where it evolved into the first major successful commercial ‘next-generation sequencing’ (NGS) technology.

**Fig. 2:**
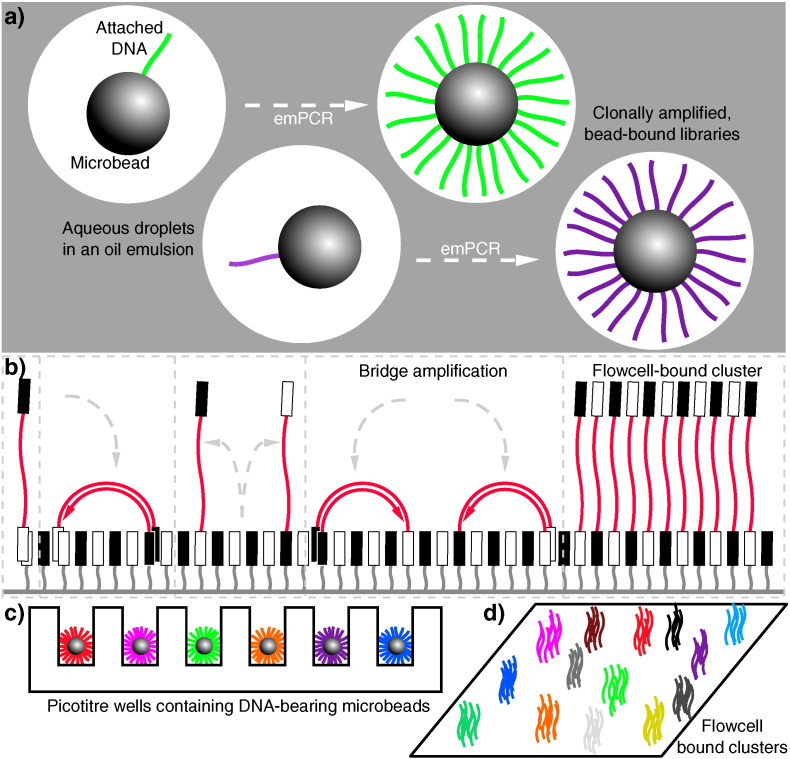
Second-generation DNA sequencing parallelized amplification. (a): DNA molecules being clonally amplified in an emulsion PCR (emPCR). Adapter ligation and PCR produces DNA libraries with appropriate 5′ and 3′ ends, which can then be made single stranded and immobilized onto individual suitably oligonucleotide-tagged microbeads. Bead-DNA conjugates can then be emulsified using aqueous amplification reagents in oil, ideally producing emulsion droplets containing only one bead (illustrated in the two leftmost droplets, with different molecules indicated in different colours). Clonal amplification then occurs during the emPCR as each template DNA is physically separate from all others, with daughter molecules remaining bound to the microbeads. This is the conceptual basis underlying sequencing in 454, Ion Torrent and polony sequencing protocols. (b): Bridge amplification to produce clusters of clonal DNA populations in a planar solid-phase PCR reaction, as occurs in Solexa/Illumina sequencing. Single-stranded DNA with terminating sequences complementary to the two lawn-oligos will anneal when washed over the flow-cell, and during isothermal PCR will replicate in a confined area, bending over to prime at neighbouring sites, producing a local cluster of identical molecules. (c) and (d) demonstrate how these two different forms of clonally-amplified sequences can then be read in a highly parallelized manner: emPCR-produced microbeads can be washed over a picotiter plate, containing wells large enough to fit only one bead (c). DNA polymerase can then be added to the wells, and each nucleotide can be washed over in turn, and dNTP incorporation monitored (e.g. via pyrophosphate or hydrogen ion release). Flow-cell bound clusters produced via bridge amplification (d) can be visualized by detecting fluorescent reversible-terminator nucleotides at the ends of a proceeding extension reaction, requiring cycle-by-cycle measurements and removal of terminators.

The sequencing machines produced by 454 (later purchased by Roche) were a paradigm shift in that they allowed the mass parallelisation of sequencing reactions, greatly increasing the amount of DNA that can be sequenced in any one run [52]. Libraries of DNA molecules are first attached to beads via adapter sequences, which then undergo a water-in-oil emulsion PCR (emPCR) [53] to coat each bead in a clonal DNA population, where ideally on average one DNA molecule ends up on one bead, which amplifies in its own droplet in the emulsion (see Fig. 2a and c). These DNA-coated beads are then washed over a picoliter reaction plate that fits one bead per well; pyrosequencing then occurs as smaller bead-linked enzymes and dNTPs are washed over the plate, and pyrophosphate release is measured using a charged couple device (CCD) sensor beneath the wells. This set up was capable of producing reads around 400–500 base pairs (bp) long, for the million or so wells that would be expected to contain suitably clonally-coated beads [52]. This parallelisation increased the yield of sequencing efforts by orders of magnitudes, for instance allowing researchers to completely sequence a single human's genome – that belonging to DNA structure pioneer, James Watson – far quicker and cheaper than a similar effort by DNA-sequencing entrepreneur Craig Venter's team using Sanger sequencing the preceding year [54], [55]. The first high-throughput sequencing (HTS) machine widely available to consumers was the original 454 machine, called the GS 20, which was later superceded by the 454 GS FLX, which offered a greater number of reads (by having more wells in the ‘picotiter’ plate) as well as better quality data [56]. This principle of performing huge numbers of parallel sequencing reactions on a micrometer scale – often made possible as a result of improvements in microfabrication and high-resolution imaging – is what came to define the second-generation of DNA sequencing [57].

A number of parallel sequencing techniques sprung up following the success of 454. The most important among them is arguably the Solexa method of sequencing, which was later acquired by Illumina [56]. Instead of parallelising by performing bead-based emPCR, adapter-bracketed DNA molecules are passed over a lawn of complementary oligonucleotides bound to a flowcell; a subsequent solid phase PCR produces neighbouring clusters of clonal populations from each of the individual original flow-cell binding DNA strands [58], [59]. This process has been dubbed ‘bridge amplification’, due to replicating DNA strands having to arch over to prime the next round of polymerisation off neighbouring surface-bound oligonucleotides (see Fig. 2b and d) [56]. Sequencing itself is achieved in a SBS manner using fluorescent ‘reversible-terminator’ dNTPs, which cannot immediately bind further nucleotides as the fluorophore occupies the 3′ hydroxyl position; this must be cleaved away before polymerisation can continue, which allows the sequencing to occur in a synchronous manner [60]. These modified dNTPs and DNA polymerase are washed over the primed, single-stranded flow-cell bound clusters in cycles. At each cycle, the identity of the incorporating nucleotide can be monitored with a CCD by exciting the fluorophores with appropriate lasers, before enzymatic removal of the blocking fluorescent moieties and continuation to the next position. While the first Genome Analyzer (GA) machines were initially only capable of producing very short reads (up to 35 bp long) they had an advantage in that they could produce paired-end (PE) data, in which the sequence at both ends of each DNA cluster is recorded. This is achieved by first obtaining one SBS read from the single-stranded flow-cell bound DNA, before performing a single round of solid-phase DNA extension from remaining flow-cell bound oligonucleotides and removing the already-sequenced strand. Having thus reversed the orientation of the DNA strands relative to the flow-cell, a second read is then obtained from the opposite end of the molecules to the first. As the input molecules are of an approximate known length, having PE data provides a greater amount of information. This improves the accuracy when mapping reads to reference sequences, especially across repetitive sequences, and aids in detection of spliced exons and rearranged DNA or fused genes. The standard Genome Analyzer version (GAIIx) was later followed by the HiSeq, a machine capable of even greater read length and depth, and then the MiSeq, which was a lower-throughput (but lower cost) machine with faster turnaround and longer read lengths [61], [62].

A number of other sequencing companies, each hosting their own novel methodologies, have also appeared (and disappeared) and had variable impacts upon both what experiments are feasible and the market at large. In the early years of second-generation sequencing perhaps the third major option (alongside 454 and Solexa/Illumina sequencing) [63] was the sequencing by oligonucleotide ligation and detection (SOLiD) system from Applied Biosystems (which became Life Technologies following a merger with Invitrogen) [64]. As its name suggests, SOLiD sequenced not by synthesis (i.e. catalysed with a polymerase), but by ligation, using a DNA ligase, building on principles established previously with the open-source ‘polony’ sequencing developed in George Church's group [65]. While the SOLiD platform is not able to produce the read length and depth of Illumina machines [66], making assembly more challenging, it has remained competitive on a cost per base basis [67]. Another notable technology based on sequence-by-ligation was Complete Genomic's ‘DNA nanoballs’ technique, where sequences are obtained similarly from probe-ligation but the clonal DNA population generation is novel: instead of a bead or bridge amplification, rolling circle amplification is used to generate long DNA chains consisting of repeating units of the template sequence bordered by adapters, which then self assemble into nanoballs, which are affixed to a slide to be sequenced [68]. The last remarkable second-generation sequencing platform is that developed by Jonathan Rothburg after leaving 454. Ion Torrent (another Life Technologies product) is the first so-called ‘post-light sequencing’ technology, as it uses neither fluorescence nor luminescence [69]. In a manner analogous to 454 sequencing, beads bearing clonal populations of DNA fragments (produced via an emPCR) are washed over a picowell plate, followed by each nucleotide in turn; however nucleotide incorporation is measured not by pyrophosphate release, but the difference in pH caused by the release of protons (H ^+^ ions) during polymerisation, made possible using the complementary metal-oxide-semiconductor (CMOS) technology used in the manufacture of microprocessor chips [69]. This technology allows for very rapid sequencing during the actual detection phase [67], although as with 454 (and all other pyrosequencing technologies) it is less able to readily interpret homopolymer sequences due to the loss of signal as multiple matching dNTPs incorporate [70].

The oft-described ‘genomics revolution’, driven in large part by these remarkable changes in nucleotide sequencing technology, has drastically altered the cost and ease associated with DNA sequencing. The capabilities of DNA sequencers have grown at a rate even faster than that seen in the computing revolution described by Moore's law: the complexity of microchips (measured by number of transistors per unit cost) doubles approximately every two years, while sequencing capabilities between 2004 and 2010 doubled every five months [71]. The various offshoot technologies are diverse in their chemistries, capabilities and specifications, providing researchers with a diverse toolbox with which to design experiments. However in recent years the Illumina sequencing platform has been the most successful, to the point of near monopoly [72] and thus can probably considered to have made the greatest contribution to the second-generation of DNA sequencers.

## 4 Third-generation DNA sequencing

There is considerable discussion about what defines the different generations of DNA sequencing technology, particularly regarding the division from second to third [73], [74], [75], [76]. Arguments are made that single molecule sequencing (SMS), real-time sequencing, and simple divergence from previous technologies should be the defining characteristics of the third-generation. It is also feasible that a particular technology might straddle the boundary. Here we consider third generation technologies to be those capable of sequencing single molecules, negating the requirement for DNA amplification shared by all previous technologies.

The first SMS technology was developed in the lab of Stephen Quake [77], [78], later commercialized by Helicos BioSciences, and worked broadly in the same manner that Illumina does, but without any bridge amplification; DNA templates become attached to a planar surface, and then propriety fluorescent reversible terminator dNTPs (so-called ‘virtual terminators’ [79]) are washed over one base a time and imaged, before cleavage and cycling the next base over. While relatively slow and expensive (and producing relatively short reads), this was the first technology to allow sequencing of non-amplified DNA, thus avoiding all associated biases and errors [73], [75]. As Helicos filed for bankruptcy early in 2012 [80] other companies took up the third-generation baton.

**Fig. 3:**
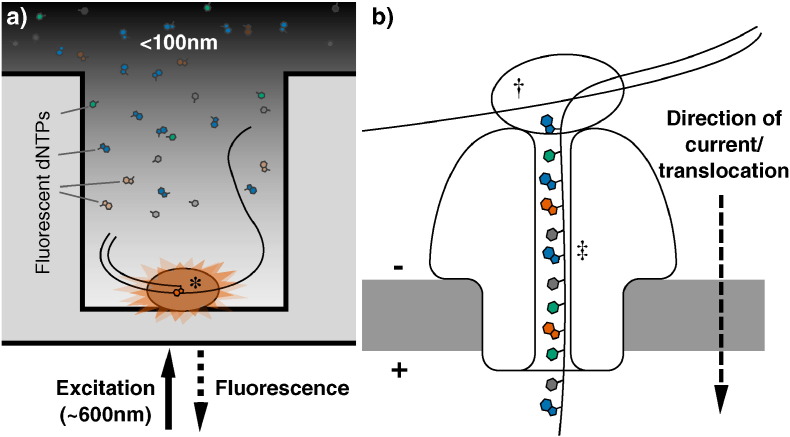
Third-generation DNA sequencing nucleotide detection. (a): Nucleotide detection in a zero-mode waveguide (ZMW), as featured in PacBio sequencers. DNA polymerase molecules are attached to the bottom of each ZMW (*), and target DNA and fluorescent nucleotides are added. As the diameter is narrower than the excitation light's wavelength, illumination rapidly decays travelling up the ZMW: nucleotides being incorporated during polymerisation at the base of the ZMW provide real-time bursts of fluorescent signal, without undue interference from other labelled dNTPs in solution. (b): Nanopore DNA sequencing as employed in ONT's MinION sequencer. Double stranded DNA gets denatured by a processive enzyme (†) which ratchets one of the strands through a biological nanopore (‡) embedded in a synthetic membrane, across which a voltage is applied. As the ssDNA passes through the nanopore the different bases prevent ionic flow in a distinctive manner, allowing the sequence of the molecule to be inferred by monitoring the current at each channel.

At the time of writing, the most widely used third-generation technology is probably the single molecule real time (SMRT) platform from Pacific Biosciences [81], available on the PacBio range of machines. During SMRT runs DNA polymerisation occurs in arrays of microfabricated nanostructures called zero-mode waveguides (ZMWs), which are essentially tiny holes in a metallic film covering a chip. These ZMWs exploit the properties of light passing through apertures of a diameter smaller than its wavelength, which causes it to decay exponentially, exclusively illuminating the very bottom of the wells. This allows visualisation of single fluorophore molecules close to the bottom of the ZMW, due to the zone of laser excitation being so small, even over the background of neighbouring molecules in solution [82]. Deposition of single DNA polymerase molecules inside the ZMWs places them inside the illuminated region (Fig. 3a): by washing over the DNA library of interest and fluorescent dNTPs, the extension of DNA chains by single nucleotides can be monitored in real time, as fluorescent nucleotide being incorporated – and only those nucleotides – will provide detectable fluorescence, after which the dye is cleaved away, ending the signal for that position [83]. This process can sequence single molecules in a very short amount of time. The PacBio range possesses a number of other advantageous features that are not widely shared among other commercially available machines. As sequencing occurs at the rate of the polymerase it produces kinetic data, allowing for detection of modified bases [84]. PacBio machines are also capable of producing incredibly long reads, up to and exceeding 10 kb in length, which are useful for de novo genome assemblies [73], [81].

Perhaps the most anticipated area for third-generation DNA sequencing development is the promise of nanopore sequencing, itself an offshoot of a larger field of using nanopores for the detection and quantification of all manner of biological and chemical molecules [85]. The potential for nanopore sequencing was first established even before second-generation sequencing had emerged, when researchers demonstrated that single-stranded RNA or DNA could be driven across a lipid bilayer through large *α*-hemolysin ion channels by electrophoresis. Moreover, passage through the channel blocks ion flow, decreasing the current for a length of time proportional to the length of the nucleic acid [86]. There is also the potential to use non-biological, solid-state technology to generate suitable nanopores, which might also provide the ability to sequence double stranded DNA molecules [87], [88]. Oxford Nanopore Technologies (ONT), the first company offering nanopore sequencers, has generated a great deal of excitement over their nanopore platforms GridION and MinION (Fig. 3b) [89], [90], the latter of which is a small, mobile phone sized USB device, which was first released to end users in an early access trial in 2014 [91]. Despite the admittedly poor quality profiles currently observed, it is hoped that such sequencers represent a genuinely disruptive technology in the DNA sequencing field, producing incredibly long read (non-amplified) sequence data far cheaper and faster than was previously possible [92], [90], [85]. Already MinIONs have been used on their own to generate bacterial genome reference sequences [93], [94] and targeted amplicons [95], [96], or used to generate a scaffold to map Illumina reads to [97], [98], [96], combining the ultra long read length of the nanopore technology and the high read depth and accuracy afforded by the short read sequencing. The fast run times and compact nature of the MinION machine also presents the opportunity to decentralize sequencing, in a move away from the core services that are common today. They can even be deployed it in the field, as proved by Joshua Quick and Nicholas Loman earlier this year when they sequenced Ebola viruses in Guinea two days after sample collection [99]. Nanopore sequencers could therefore revolutionize not just the composition of the data that can be produced, but where and when it can be produced, and by whom.

## 5 Conclusions

It is hard to overstate the importance of DNA sequencing to biological research; at the most fundamental level it is how we measure one of the major properties by which terrestrial life forms can be defined and differentiated from each other. Therefore over the last half century many researchers from around the globe have invested a great deal of time and resources to developing and improving the technologies that underpin DNA sequencing. At the genesis of this field, working primarily from accessible RNA targets, researchers would spend years laboriously producing sequences that might number from a dozen to a hundred nucleotides in length. Over the years, innovations in sequencing protocols, molecular biology and automation increased the technological capabilities of sequencing while decreasing the cost, allowing the reading of DNA hundreds of basepairs in length, massively parallelized to produce gigabases of data in one run. Researchers moved from the lab to the computer, from pouring over gels to running code. Genomes were decoded, papers published, companies started – and often later dissolved – with repositories of DNA sequence data growing all the while. Therefore DNA sequencing – in many respects a relatively recent and forward-focussed research discipline – has a rich history. An understanding of this history can provide appreciation of current methodologies and provide new insights for future ones, as lessons learnt in the previous generation inform the progress of the next.
